# Emotional words can be embodied or disembodied: the role of superficial vs. deep types of processing

**DOI:** 10.3389/fpsyg.2015.00975

**Published:** 2015-07-09

**Authors:** Ensie Abbassi, Isabelle Blanchette, Ana I. Ansaldo, Habib Ghassemzadeh, Yves Joanette

**Affiliations:** ^1^Centre de Recherche, Institut Universitaire de Gériatrie de Montréal and Faculté de Médecine, Université de Montréal, Montréal, QCCanada; ^2^Département de Psychologie, Université du Québec à Trois-Rivières, Trois-Rivières, QCCanada; ^3^Department of Psychiatry, Tehran University of Medical Sciences, TehranIran; ^4^Visiting Scholar, University of Oregon, Eugene, ORUSA

**Keywords:** emotional words, meaning access, conceptual processing, disembodied/embodied, superficial/deep, cerebral hemispheres

## Abstract

Emotional words are processed rapidly and automatically in the left hemisphere (LH) and slowly, with the involvement of attention, in the right hemisphere (RH). This review aims to find the reason for this difference and suggests that emotional words can be processed superficially or deeply due to the involvement of the linguistic and imagery systems, respectively. During superficial processing, emotional words likely make connections only with semantically associated words in the LH. This part of the process is automatic and may be sufficient for the purpose of language processing. Deep processing, in contrast, seems to involve conceptual information and imagery of a word’s perceptual and emotional properties using autobiographical memory contents. Imagery and the involvement of autobiographical memory likely differentiate between emotional and neutral word processing and explain the salient role of the RH in emotional word processing. It is concluded that the level of emotional word processing in the RH should be deeper than in the LH and, thus, it is conceivable that the slow mode of processing adds certain qualities to the output.

## Introduction

This paper concerns emotional words and how these words are processed in the cerebral hemispheres. Our previous review (Abbassi et al., 2011), based on behavioral, electrophysiological, and neuroimaging research results, indicates that both hemispheres are involved in the processing of emotional words, albeit in different and probably complementary ways. Emotional words are processed rapidly early in processing, and slowly with the involvement of attention later on; the left hemisphere (LH) and the right hemisphere (RH) are likely responsible for this early vs. later stage of processing, respectively^1^. Automatic processing does not place much demand on processing resources whereas attentional processing is slow, effortful, and under one’s active control. This paper aims to pinpoint the nature of emotional word processing and find the reason for the rapid vs. slow modes of processing. It shows that emotional word processing does not necessarily produce a subjective experience of emotions (though it may); thus, a kind of superficial processing is also possible and is probably the reason for fast and automatic processing of these words in the LH. Deep kind of processing, in contrast, is likely the reason for slow processing of emotional words in the RH.

By emotional word, we refer to any word characterized by emotional connotations (e.g., “lonely,” “poverty,” “neglect,” “bless,” “reward,” “elegant”) or denoting a specific emotional reaction (e.g., “anger,” “happy,” “sadness“). Although emotional words can convey the emotions we feel, they can be used without subjective experiencing of an emotion, as well (see Niedenthal et al., 2003, for a review).We have non-verbal channels – facial expression, prosody, and body language – to communicate emotions. So is there another reason for using emotional words? How do emotional words convey emotional meanings? What underlies the rapid^2^ vs. slow modes of processing of emotional words? To answer these questions, it is necessary to first explore the purpose of using the language system and its relationship with semantic memory, where meanings and concepts, or knowledge about the world, are represented in the mind. This understanding is fundamental to an understanding of emotional words and concepts and how these words are processed in the cerebral hemispheres. Then we can describe the role that emotional words play in human communication and the reason for the automatic vs. attentional modes of processing of emotional words in the LH and RH.

Accordingly, in the first section of the paper, we present two approaches – disembodied and embodied – to concept representation and meaning access and then an integrative approach that combines the capabilities of both. Next, the linguistic and simulation (i.e., perceptual- or image-based) systems, which are involved in conceptual processing and meaning access, are presented. By the linguistic system here we refer to the system for which linguistic forms^3^ are also important; thus, meaning is mainly represented in the simulation system (Barsalou et al., 2008). After that, we describe research results that show how conceptual processing and meaning access occur using the linguistic and simulation systems. Since emotional words are generally more abstract, we will then discuss the discriminating features of abstract and concrete words. Then, we present evidence demonstrating that meaning access during the early, automatic processing of emotional words is superficial and is accomplished by the linguistic system, whereas meaning access during the later attentional processing of emotional words is deep, and involves imagery and the content of autobiographical memory. The paper will end with a proposed framework that attributes the superficial mode of emotional word processing to the LH and the deep mode to the RH.

## Meaning Access and Conceptual Processing

### Semantic Memory and Approaches to Concept (Meaning) Representation

We store knowledge we have acquired about the world, including concepts, facts, skills, ideas, and beliefs, in a division of long-term memory known as *semantic memory* (Glaser, 1992; Martin, 2001; Thompson-Schill et al., 2006). Because concepts play important roles in different cognitive operations, semantic memory is sometimes known as conceptual memory or the conceptual system (e.g., Barsalou, 2003b). Unlike *episodic memory*, which is a person’s unique memory of events and experiences (e.g., times, places), semantic memory consists of memories shared by members of a culture (Tulving, 1972, 1984). For example, whereas remembering the name and breed of our first dog is dependent on episodic memory, knowing the meaning of the word “dog”^4^ and what a dog is relies on semantic memory. Thus, studying semantic memory and conceptual processing is a window that guides us toward the way in which word meanings are accessed. When we have a concept for something, it means that we know its meaning. How is a concept stored in semantic memory? What is the nature of the concepts or meanings stored in the mind? Cognitive literature introduces two main approaches in this respect: *disembodied* or *symbolic* (amodal) and *embodied* (modal).

#### Disembodied Approach

The symbolic approach, which corresponds to the more traditional view, assumes that there is no similarity between components of experience – objects, settings, people, actions, events, mental states, and relations – and concepts stored in the mind. This approach proposes that perceptual –sensory, motor, introspective (e.g., mental states, affective, emotional^5^) – information about components of experience is transduced (re-described) into arbitrary (language-like) symbols such that the final concept contains no reference to the actual experience *per se* (e.g., Fodor, 1975; Pylyshyn, 1984; see Barsalou and Hale, 1993, for review). Thus, abstract symbolic codes constitute concepts and perceptual experiences do not play a role in knowledge representation. Semantic networks which represent semantic relations between concepts constitute one example of this mode of knowledge representation (Collins and Loftus, 1975; Posner and Snyder, 1975).

#### Embodied Approach

The last two decades have witnessed a surge of interest in alternative models of concept representation clustered under the label *embodied* or *modal* approach (e.g., Barsalou, 1999; Wilson, 2002; Gallese and Lakoff, 2005; Glenberg, 2010). This approach assumes that various sensory (visual, auditory, tactile, etc.), motor, and introspective information about external world experiences is depicted in the brain’s modality-specific systems. Indeed, the claim that concepts are embodied means that they are formed as a result of interactions with objects, individuals, and the real world as a whole in modality-specific brain areas that are responsible for processing the corresponding perceptual information (Zwaan, 2004).

Thus, when an entity (e.g., object, event) is experienced, it activates neurons in the sensory, motor, and affective neural systems. For example, when one sees a car, a group of neurons fires for color, others for shape, a third group for size, and so forth, to represent CAR in one’s vision. Regarding the auditory and tactile sensory modalities, analogous patterns of activation can occur to represent how a car might sound or feel. Moreover, activation of neurons in the motor system represents actions on the car, and activations that occur in emotion-related areas like the amygdala and orbitofrontal regions represent emotional reactions toward the car (Barsalou, 1999, 2003a, 2008, 2009). So concepts have the same structure as perceptual experiences.

#### Integrative Approach

The cognitive literature has recently appeared to provide support for a middle approach to concept representation that combines the two approaches described above (e.g., Vigliocco et al., 2004; Barsalou et al., 2008; Louwerse, 2008, 2011; Simmons et al., 2008; Louwerse and Hutchinson, 2012). Indeed, considering the organization of the nervous system, it is hard to accept either the pure disembodied or the pure embodied approach. The nervous system has not only modality-specific (unimodal) areas but also supramodal areas. This middle position proposes that concept representation involves some form of symbolic information, along with the activation of sensory, motor, and emotional areas (e.g., Machery, 2007; Mahon and Caramazza, 2008; Meteyard et al., 2012). To be specific, meaning is both grounded in relation between words and in perceptual experiences (e.g., Vigliocco et al., 2004, 2009). There might, however, be important differences; when meaning access is the product of relation between words, it could be thought of as superficial while meaning access resulting from activating perceptual experiences may be deeper, an idea we come back to later.

Binder and Desai (2011) suggest the term *embodied abstraction*, which means that conceptual representation consists of several levels of abstraction from sensory, motor, and emotional input. The top level is highly abstract and activation in this level is sufficient for familiar (already categorized) processes such as lexical decision tasks (Glaser, 1992). Consequently, processing does not involve activation of modality-specific areas. In contrast, when a deep type of processing is necessary or possible, such as when the exposure duration of words is long (e.g., Simmons et al., 2008), perceptual areas play a greater role in performing a task.

Based on this integrative approach, we can expect words to first create activation in supramodal areas that are not modality-specific, in areas such as the anterior temporal lobe, which has been described as the neural substrate behind semantic memory (Simmons and Barsalou, 2003; Kiefer et al., 2007a,b; Patterson et al., 2007; Mahon and Caramazza, 2008; Pulvermüller et al., 2010). Activation in these areas is typically left-lateralized, whereas bilateral activation can be expected when perceptual areas come into play (e.g., Van Dam et al., 2010). This approach likely implies two levels of processing: one level that is rather superficial and another level that is deep and during which activation spreads to modality-specific areas (e.g., visual cortex, auditory cortex, motor cortex). The main point here is that word processing relies on both amodal and modality-specific areas.

### Two Systems Involved in Conceptual Processing and Meaning Access

Most researchers working in the fields related to conceptual processing accept that two systems – a language like system (i.e., linguistic system) and a perceptual- or image-based system (i.e., simulation) – are involved in conceptual processing (e.g., Paivio, 1971, 1986, 1991; Glaser, 1992; Barsalou, 2008). A detailed investigation of the role of these two systems is found in Barsalou’s (2008) LASS theory – linguistic and situated simulation. According to this theory, the linguistic system helps us communicate the concepts that we have stored in our mind and create a network containing semantically associated words. This network encompasses categories of words and relations among concepts.

Simulation or reenactment is the process by which concepts re-evoke or produce perceptual states present when perceiving and acting in the real world. In other words, our perceptual system can become active in the absence of external world entities. Researchers consider simulation to be the factor that supports the spectrum of cognitive functions from perception to thought and reasoning (Barsalou et al., 1999, 2003; Martin, 2001; Barsalou, 2003b). For example, being able to name the different colors of an APPLE (red, yellow, green) is possible due to simulation. Simulation is also considered to be *situated* (Barsalou, 2003b; Yeh and Barsalou, 2006). That is, during simulation not only the target object (e.g., APPLE) is simulated, but also settings, actions, and introspections. This triggers an experience of *being there*. Thus, when an APPLE is simulated, it occurs in a setting like a garden, with apples hanging from the branches of a tree, with someone eating it, probably experiencing a pleasant taste.

**FIGURE 1:**
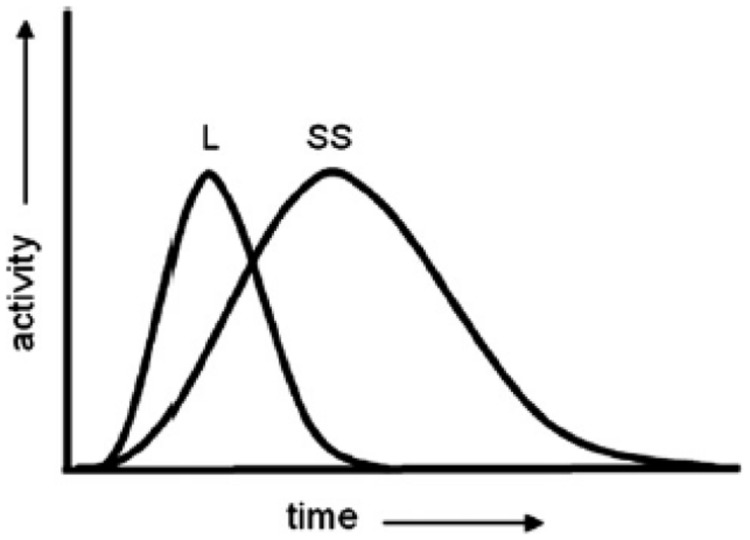
**The LASS theory – linguistic and situated simulation: initial contributions from the linguistic system (L) and the situated simulation (SS) system during conceptual processing.** When the cue is a word, contribution from the linguistic system precedes those from the simulation system. The height, width, shape, and offset of two distributions are not assumed to be fixed. In response to different words in different task contexts, all these parameters are expected to change (e.g., SS activity could be more intense than L activity). Thus, the two distributions in this figure illustrate one of infinitely many different forms that activations of the L and SS systems could take. Figure and legend reproduced with permission from Barsalou et al. (2008).

As the **Figure 1** illustrates, the LASS theory holds that when a word is presented (heard or seen), both the linguistic and simulation systems become active immediately to access its meaning; however, the activation of the linguistic system peaks before that of the simulation system. The reason is probably that the linguistic forms of representation are more analogous to the perceived words than the simulation of related experiences. Barsalou et al. (2008) claimed that, although the simulation system existed long before human beings evolved, the use of the linguistic system was what caused humans to enhance their cognitive performance. It is as though the linguistic system appeared later in human development in order to control the simulation system and increase this system’s ability to represent non-present situations.

Yet Barsalou et al. (2008) proposed that the activation of the linguistic system is rather superficial because meaning is principally represented in the simulation system. For example, “car” first activates “vehicle” and “automobile” and then these associated linguistic forms act like pointers to related conceptual information. This process causes simulation to occur and processing to become deep and deeper. Because the simulation of related conceptual information proceeds more slowly than the activation of associated words, the linguistic stage peaks earlier than the simulation stage.

Research shows different combinations of the activity of the linguistic and simulation systems underlie a wide variety of tasks. When a superficial mode of processing is sufficient for adequate task performance, processing is supported mostly by the linguistic system and little by the simulation system. In contrast, when the linguistic system cannot complete a task on its own or there is opportunity (more time) for additional processing, attention shifts to the simulation system which takes extra time.

#### Evidence for Mixtures of Linguistic and Simulation Systems in Conceptual Processing and Meaning Access

The cognitive science literature provides evidence for a superficial mode of processing managed by the early acting linguistic system and a deep mode of processing managed by the later-acting simulation system. Two tasks have provided evidence for this difference in depth of processing: the property verification task and the property generation task. We next describe the results of research employing these two tasks, including one recent study using event-related potentials (ERPs), to further understand the nature of conceptual processing.

##### Evidence from the property verification task

A property verification task (e.g., Solomon and Barsalou, 2001, 2004; Kan et al., 2003; Pecher et al., 2003; Van Dantzig et al., 2008) is a passive, recognition-oriented task, in which the participant reads a concept word (e.g., an object name such as “chair”) presented on a computer screen and verifies whether the next presented word is a true or false property of that concept (e.g., “facet” vs. “seat”). Response time and accuracy are measured. Typically, the simulation system is expected to be involved in responding to this task. That is because conceptual information must be retrieved that identifies whether the property is a part of the concept. An interesting finding is that when the property of a target trial (e.g., LEMON-“sour”) relates to a different modality than the previous trial (e.g., BLENDER-“loud”), switching occurs between sensory modalities. This incurs a processing cost: slower and less accurate responses (Pecher et al., 2003) because attention must switch from one modality to another.

Nevertheless, task condition may cause participants to mostly rely on the linguistic system. That is, when information in the linguistic system is sufficient, participants do not utilize the simulation system (Solomon and Barsalou, 2004). On true trials, the given property is always part of the concept (e.g., ELEPHANT-“tusk,” SAILBOAT-“mast”). Consequently, the type of false properties presented is the factor that determines whether the processing is superficial or deep. On false trials, if the given property is unrelated to the concept (e.g., AIRPLANE-“cake,” BUS-“fruit”), the involvement of the linguistic system is sufficient for adequate performance. That is because correct responses in this condition are highly correlated with linguistic associativeness; i.e., object and property being associated is equal to a *true* response and being not associated is equal to a *false* response. Thus, participants consult only the linguistic system and processing is superficial.

In contrast, when true trials (e.g., ELEPHANT-“tusk,” SAILBOAT-“mast”) are mixed with false trials in which the property is associated to the concept but is not a part of its concept (e.g., TABLE-“furniture,” BANANA-“monkey”), consulting the linguistic system is not sufficient. Consequently, participants must simulate perceptual information for adequate performance. Therefore, research shows participants are quite faster (more than 100 ms) to verify the same true trials when the false trials are unrelated than when they are related (Solomon and Barsalou, 2004). This is evidence that the linguistic system can act faster and produce responses earlier than the simulation system.

##### Evidence from the property generation task

A property generation task (e.g., Wu and Barsalou, 2009; Santos et al., 2011) is an active, production-oriented task, in which a word for a concept (e.g., “table”) is presented to the participant who is asked to verbally generate its characteristic properties (e.g., “legs,” “surface,” “eating on it”). This task is an important tool in the psychology of concepts; it provides a window into the underlying representation of a concept. The properties that participants produce can reveal which system is involved in meaning access. Since deep retrieval of a concept involves simulation, experts in concepts believe that property generation involves perceptual representation.

In a series of experiments, Santos et al. (2011) gave participants words like “car,” “bee,” “throw,” and “good,” and asked for the following word, that is, what other words came to mind immediately. The words produced in a 5-s period (usually 1–3 words) were analyzed. Analysis of the responses showed the linguistic origins of the various words produced such as *compounds* (e.g., the response “hive” to “bee” comes from “beehive”), *synonyms* (e.g., “automobile” in response to “car”), *antonyms* (e.g., “bad” in response to “good”), *root similarity* (e.g., “selfish” in response to “self”), and *sound similarity* (e.g., “dumpy” in response to “lumpy”).

In contrast, when participants were asked what characteristics are typically true of (for instance) “dogs” and responses given during a 15-s period were analyzed, most of the words produced originated in the simulation system. In fact, the first responses were still linguistic-based, but they were followed by responses originating in the simulation system. Thus, later responses described aspects of situations such as *physical properties* (e.g., “wings” in response to “bee”), *setting information* (e.g., “flowers” in response to “bee”), and *mental states* (e.g., “boring” in response to “golf”). Overall, the results suggest that both a faster-acting linguistic system and a slower-acting simulation system are involved in conceptual processing.

Similar findings were obtained when Simmons et al. (2008) administered a property generation task in an MRI scanner. In the first session, participants generated the typical properties of each concept to themselves for 15 s (property generation task), and in the second session, they were given some other words and generated *word associates* for each one for 5 s (word associate task). In this session, for six presented words, participants were given 15 s to imagine a situation that contained the related concept [situated simulation (SS) task]. For example, for BEE they might imagine a garden with a bee buzzing around flowers and then flying toward a hive and so forth.

To analyze the data, each 15-s period of response time for a single word was divided into two 7.5-s periods, an early one and a later one. The results showed three regions of overlap between the early stage of the property generation task and the word association task: Broca’s area (the left inferior frontal gyrus), left inferior temporal gyrus, and right cerebellum. These regions are responsible for linguistic processing and generating two-word associations (right cerebellum). A different set of regions overlapped between the later stage of the property generation task and the SS task: bilateral posterior areas, precuneus, right middle temporal gyrus, and right middle frontal gyrus. These regions are involved in imagery, episodic memory, and situation representation. Thus, fMRI research corroborates findings from behavioral studies: properties bearing a linguistic relation to presented words were produced earlier than properties bearing a simulation relation.

##### Evidence from time course analysis

A recent ERP investigation conducted by Louwerse and Hutchinson (2012) also suggests different time course of activation for linguistic and simulation processing, with evidence that the linguistic system reaches its peak of activation earlier that the simulation system. Half of the participants were assigned to a semantic judgment task and the other half to an iconicity judgment task. Each condition employed the same word pairs, half with an iconic relationship in which the two words were presented vertically in the same order that they appear in the world (e.g., “sky” above “ground”) and the other half with a reverse-iconic relationship (e.g., “ground” above “sky”). In the semantic task, participants judged whether the words were related in meaning; in the iconicity task, participants judged whether the words appeared in the same configuration as in the real world (i.e., a yes/no response was required for both tasks).

The results showed the involvement of both linguistic and simulation systems in responding to both tasks, with the linguistic system being more active during the semantic task and the simulation system during the iconicity task. Participants took a mean 1809 ms to respond to task stimuli. Source analysis using Low Resolution Brain Electromagnetic Tomography (LORETA) showed that, for each trial, activation started (within 300 ms after stimulus onset) around the left inferior frontal gyrus (FC5, F7, T7) and then continued (within 1500–1800 ms after stimulus onset) bilaterally in posterior areas of the brain (O1, O2, P7, P8).

Overall, research findings suggest that, when words are presented, two systems of linguistic and simulation come into play immediately to access their meanings. However, the activation in the linguistic system peaks early on; this type of processing is rather superficial and involves activation of a word’s associates in the LH. In contrast, the activation in the simulation system peaks later; this type of processing is deep and involves SS of the referent of a word, for which bilateral activation may be required.

### Abstract vs. Concrete Word Processing

A key topic of discussion concerning word and concept processing relates to abstract words. This is important because abstract words, on average, tend to have more emotional properties than concrete words (Kousta et al., 2011; Moseley et al., 2012; Sakreida et al., 2013; see Meteyard et al., 2012, for a review). Hence, we need to discuss the differentiating characteristics of abstract words. Some concept experts (e.g., Paivio, 1971, 1986) claim that abstract concepts are represented only through associations with other words, that is, through the linguistic system. This notion may have been reinforced by the results of neuroimaging research, which demonstrate more activation of the LH during the processing of abstract words compared to concrete words (see Sabsevitz et al., 2005, for a review).

However, Barsalou (1999) casts doubt on this notion and attributes this notion to the kind of task generally used to study abstract words. He believes that abstract words cannot be learned without the contribution of SSs. In effect, abstract concepts are represented in a wide variety of situations featuring predominantly introspective (emotional) and social information, whereas concrete concepts are represented in a restricted range of situations featuring chiefly sensory and motor information. This difference causes the situation to play a critical role in deep processing of abstract words. That is to say, both linguistic and simulation systems are both involved in the processing of abstract words; nevertheless, the type of task determines whether the linguistic or simulation system is active.

In one study conducted by Barsalou and Wiemer-Hastings (2005) a property generation task was used to compare representations of abstract concepts (e.g., TRUTH, FREEDOM, INVENTION) and concrete concepts (e.g., BIRD, CAR, SOFA). Simulation was shown to be also important in the representation of abstract concepts. When participants were asked to generate properties of concrete and abstract concepts, in both cases, they produced relevant information about agents, objects, settings, events, and mental states. However, the emphasis placed on these different types of information was different. For concrete concepts, the major focus was on information about *objects* and *settings*. In contrast, for abstract concepts, more information about *mental states* and *events* was produced.

As well, neuroimaging research demonstrates that abstract concepts are represented by distributed neural patterns more than concrete concepts (Barsalou and Wiemer-Hastings, 2005; Wilson-Mendenhall et al., 2011; Moseley et al., 2012). Thus, for an abstract word like “convince,” a variety of situations (e.g., a political situation, a sports situation, a school situation, etc.) may come to mind to represent events in which one person (agent) is speaking to another or others in order to change their mind. It seems to be difficult for people to process an abstract word without bringing relevant situations into their mind (Schwanenflugel, 1991). For a concrete word like “rolling,” in contrast, the processing is simpler and more focused. Thus, the role that a task plays is critical here: if the researcher was using a lexical decision task, which typically encourages a superficial level of processing, there is more possibility that an abstract word will access only the information provided by the linguistic system (Glaser, 1992; Kan et al., 2003; Solomon and Barsalou, 2004). The involvement of the simulation system, on the other hand, requires a task that encourages deep processing (Wilson-Mendenhall et al., 2013).

This paper concerns emotional words, many of which are abstract. So we can predict that the above descriptions of word and concept processing, which show words can be processed superficially or deeply, will apply to emotional words and concepts, as well. A review of the findings related to emotional word processing should help verify this prediction.

## Emotional Word Processing and Meaning Access

In the second part of this paper we focus on emotional word processing. We intend to show that, similarly to what we have established thus far for neutral words, a superficial type of processing also occurs for emotional words. Although emotional words possess emotional component, their processing does not necessarily results in emotional states (e.g., Innes-Ker and Niedenthal, 2002; Havas et al., 2007). That is, creating emotional states and communicating emotional feelings may not be the only or primary outcome of using emotional words. We have other channels – prosody, facial expression, and body language – to communicate our feelings. That is why some researchers believe that language is a tool by which we can control our emotions (see Niedenthal et al., 2003, for a review). The linguistic system, which underlies superficial word processing, likely leads to conveying information about emotions without necessarily experiencing emotional states. We suggest that the involvement of an image-based system is necessary to experience emotional states as a result of emotional word processing.

Thus we suggest that, similarly to what occurs for neutral words, the involvement of a perceptual- or image-based system is likely necessary for deep processing of emotional words. There is, however, one important difference between deep processing of neutral and emotional words. In deep processing of emotional words, emotional properties^6^ likely have a crucial influence on the outcome of the processing. This notion does not deny the important role that perceptual (i.e., visual, auditory, etc.) properties play in this process. As a result, we can imagine deep processing of emotional words which may involve reactivation^7^ of emotional properties, to be able to create emotional states in the individual (e.g., Havas et al., 2007). This does not mean that deep processing is always along with reactivation of emotional properties, but it keeps open different possibilities for the reactivation of emotional and perceptual properties: for example, reactivation of only perceptual properties without emotional properties, reactivation of one perceptual property (for instance, visual) along with emotional properties, etc.

Therefore, the principal question in this part is: what does happen when emotional words are presented? In the following sections, after introducing emotional concepts, related approaches, and an overview of the lateralization of emotional word processing, we attempt to answer this question and find the reason for rapid vs. slow modes of processing of emotional words in the cerebral hemispheres.

### Emotional Concepts

Emotional concepts (e.g., FIGHT, SPIDER, JOY, ANGER) represent knowledge about emotions, that is, the meaning of emotional information. They hold information about behaviors associated with emotions (e.g., actions), how emotions are elicited (e.g., situations), and subjective experiences and bodily states that occur when we are in an emotional state (Niedenthal, 2008). Based on the information presented above, there should be a link between emotional words and emotional concepts, i.e., emotional words should serve as a window to access emotional concepts. Reactivating the emotional properties of emotional concepts can be expected, in turn, to lead to subjective emotional states.

Regarding the nature of emotional concepts, the literature on emotion, perhaps under the influence of cognitive studies, has adopted two approaches: disembodied or amodal and embodied or modal. In the disembodied approach (Teasdale, 1999; Philippot and Schaefer, 2001), emotions are represented in an amodal fashion, devoid of their perceptual and emotional properties. Namely, emotional information that is initially encoded in different modalities (i.e., visual, auditory, etc.) is represented and stored in the conceptual system separate from its perceptual and emotional properties. Thus, just as people know that CHAIR possesses the properties of seat, back, and legs, they know that ANGER comprises the experience of frustration, a desire to fight, maybe a clenched fist, and a rise in blood pressure.

On the other hand, the embodied or modal approach proposes that sensory, motor, and emotional states triggered during an encounter with an emotion-evoking stimulus (e.g., a SNAKE) are captured and stored in modality-specific brain areas (Damasio, 1989; Damasio and Damasio, 1994; Gallese, 2003; Niedenthal et al., 2005a,b; Barrett, 2006). Later, during reactivation of the experience (e.g., thinking about a snake), the original pattern of sensory, motor, and emotional states can be relived. More specifically, emotional states (e.g., feeling happy, sad, angry) that are experienced during interaction with stimuli having pleasant or unpleasant properties are stored and later reactivated. Thus, like other concepts, processing of emotional concepts is accompanied by reactivation of subjective experiences in modality-specific areas in the brain (Chao and Martin, 2000; Pecher et al., 2003; Vermeulen et al., 2007).

Similar to word and concept processing in general, the literature provides evidence for rapid simultaneous activation of the linguistic and emotional areas in the LH when emotional words are presented (e.g., Herbert et al., 2011; Moseley et al., 2012; Ponz et al., 2013; see Abbassi et al., 2011, for review), in addition to a slower activation that occurs later on in the RH. Our previous review (Abbassi et al., 2011) suggests that during the automatic processing of emotional words, in tasks such as lexical decision where deep processing is not required, early ERP components like early posterior negativity (EPN)^8^ which occur within 300 ms of stimulus onset, appear. For this type of processing, in addition to language areas including the inferior frontal (Broca’s area), inferior parietal, and superior temporal (Wernicke’s area), limbic areas including the orbitofrontal, prefrontal, amygdala, posterior cingulate, and insular cortex are also activated.

In contrast, when an explicit task like emotional Stroop task^9^ is used, later ERP components like late positive component (LPC) that occur more than 300 ms from stimulus onset, appear and processing is stronger in the RH. This type of processing is slow, requires attention, and also the involvement of some other areas including anterior cingulate and dorsolateral prefrontal cortex (Abbassi et al., 2011, for review). Accordingly, the same as neutral word processing, it appears that emotional word processing requires two systems: the linguistic system which peaks early on and a second system, we will introduce it shortly, which (like simulation) involves image reproduction, but with some specificities relative to neutral words. The results of the relevant research introducing the capabilities of these two systems follow.

### Two Levels of Emotional Word Processing

#### Superficial Level

Research suggests that emotional word processing is not always accompanied by feeling an emotion. Indeed, an emotional word can be processed superficially, such that no subjective experience of emotion (emotional feeling) becomes involved. That is to say, although emotional words possess emotional properties, their processing does not necessarily lead to feeling an emotion.

One task that demonstrates this finding is the *sentence unscrambling task* (e.g., Srull and Wyer, 1979; Bargh et al., 1996; Innes-Ker and Niedenthal, 2002; Oosterwijk et al., 2010). In this task, participants are presented with a series of words in random order and asked to construct grammatically correct sentences out of a subset of the words. Critical sentences are intended to prime a specific pleasant or unpleasant concept (e.g., HAPPINESS, SADNESS). For example, in the study conducted by Innes-Ker and Niedenthal (2002), 30 four-word sentences that described behaviors, situations, and reactions associated with happy or sad feelings were used. A fifth word was added to each sentence to create groups of five scrambled words. The connotation of this word was the same as the sentence (e.g., “the guest felt satisfied” as the sentence and “ease” as the filler). Fifteen sentences with neutral content were also added to each list to control for the bias in favor of the intended emotional concept. Participants were asked to construct a four-word sentence out of each subset.

After performing the task, participants completed a self-report measure of emotional state and also a lexical decision experiment. The results indicated that unscrambling emotional sentences did not affect participants’ emotional state. Yet, performing the task was effective in priming semantically related words having emotional component, because participants made faster lexical decisions about words that were congruent with the activated concept than about incongruent words (e.g., “joke” primed “sunbeam,” not “speech”; “tears” primed “disease,” not “breath”). Thus, participants could encounter emotional words and construct sentences with an emotional meaning, but without reactivating that meaning sufficiently to trigger an emotional subjective experience.

Here, we need to make a distinction between high-level subjective emotional experience that we refer to in this paper and low-level affective or arousal changes that seem to occur automatically early in processing (e.g., Kissler et al., 2007). Although the sentence unscrambling task does not evoke subjective emotional states, research shows it can evoke low-level affective changes in the body and face (Oosterwijk et al., 2010). This type of (low-level) changes likely gives intensity to our subjective experiences, i.e., what we feel later on^5^. Thus, following Barsalou et al. (2008), we believe two systems (linguistic and image-based) are activated when emotional words are presented. The linguistic system, however, peaks before the image-based system. That is, during superficial processing of emotional words, in addition to word forms (linguistic system), arousal features are also accessed; these features likely potentiate subsequent emotional feelings (Oosterwijk et al., 2010; Citron, 2012; Citron et al., 2014).

So emotions are not necessarily experienced when we encounter emotional words. That is probably because emotional words are processed only superficially at first. In order to feel an emotion, it appears that the brain areas responsible for emotion must reactivate an emotional experience. Accordingly, Niedenthal et al. (1994) suggested that emotional knowledge is represented at three levels. The first is the *emotion lexicon level*, which includes words; this level is necessary for *encoding* perceptual and emotional experiences. The second level is the *conceptual level*, which contains memories of emotional experiences. The third level is the *somatic level*; at this level, feedback from the body is recognized and bodily changes affecting the autonomic nervous, endocrine, and muscular systems are experienced^10^.

Therefore, reactivation of emotional states is likely a prerequisite for subjective experiencing of an emotion; activation of associated words does not lead to emotional feelings. If we know that concepts are grounded not only in sensory and motor experiences but also in emotional experiences, we can expect reactivation of emotional experiences, similar to the reactivation of sensory and motor experiences, to occur (Barsalou, 1999).

#### Deep Level

##### Evidence from the property verification task

Researchers working in areas related to emotion have used the property verification task to show that the emotional properties of concepts can be reactivated using the same system that supports emotional responses to an object or event. For example, in Vermeulen et al.’s (2007) study, not only perceptual (e.g., visual, auditory) but also emotional (pleasant and unpleasant) properties of concepts were taken into consideration. Half of the trials were constructed of concepts paired with properties coming from the same modality as in the previous trial (e.g., TRIUMPH-“exhilarating”/COUPLE-“happy”) and the other half of concepts paired with properties coming from different modalities than in the previous trial (e.g., FRIEND-“tender”/TREASURE-“bright”). In such a task, verifying, for instance, that TRIUMPH can be “exhilarating” or a COUPLE can be “happy” involves reactivating emotional properties in the emotional system whereas verifying that a FRIEND can be “tender” after verifying that a TREASURE can be “bright” reactivate two different systems (the emotional system for the former and the visual system for the later).

Thus, similar to the switching cost for neutral pairs that we reviewed earlier (e.g., Pecher et al., 2003), the results showed slower reaction times and higher error rates when judgments required participants to switch modalities, that is, when trials with emotional properties were preceded by trials with perceptual properties. This finding suggests that, in order to verify an emotional property, this property needs to be reactivated by the emotional system.

##### Evidence from the property generation task

Property generation tasks have yielded the same conclusion as property verification tasks. Since emotional words are more abstract, we would expect participants to generate and focus on situations, events and introspective (including emotional) properties when a property generation task is used (Martin and Chao, 2001). Accordingly, when Oosterwijk et al. (2009) asked participants to generate properties of the emotional words “pride” and “disappointment,” most of the generated words described situations, personal attributions, events, and associated reactions rather than agents and objects.

For “pride,” words or phrases like “school,” “sport,” “good marks,” “winning a game,” “did well,” “applause,” “throwing a party,” “feeling happy,” and “significant others (parents, friends, family)” were produced. Likewise, for “disappointment,” words or phrases like “doing badly,” “losing,” “failing psychophysiology,” “getting an F,” “exams,” “driving test,” “shame,” “fear,” “feeling angry,” and “depression” were generated. As indicated, almost all the words generated referred to situations, actions, and introspective states, and many referred directly to emotional states. Producing words or phrases like “feeling happy” even suggests the possibility that the participant may experience an emotion (Barsalou, 1999).

One point that merits special attention and seems to be indicated by the results is the likely activation of autobiographical memory when emotional words are processed. Here, we need to consider the differentiating feature of autobiographical memory and episodic memory. In fact, some researchers treat autobiographical and episodic memory as synonyms, but others believe they should be treated separately (e.g., Tulving, 1983; Conway, 1990; Cabeza et al., 2004; McDermott et al., 2009; see Marsh and Roediger, 2012, for a review). They say that memory for events with specific times and places should be referred to as episodic memory, whereas autobiographical memory is related to our personal history in which priorities are given to emotional properties, not to specific times and places: memories, for instance, of our first-grade experiences, of learning to drive a car, of friends we had in university, or of grandparents. Conway (1990) argues that autobiographical memory plays a pivotal role in the representation of emotional information.

In the next section, we discuss autobiographical memory which seems to provide the content for imagery^11^ system and whose role in emotional word processing is likely comparable with semantic memory which provides content for the simulation system. While literature suggests that semantic memory is likely centered in the LH, the concentration of autobiographical memory seems to be in the RH (see Cabeza and Nyberg, 2000, for a review). We suggest this system is crucially involved in deeper processing of emotional words.

### Imagery, Autobiographical Memory, and the RH

As mentioned above, reactivation (simulation) of stored information appears to be necessary for deep processing of word stimuli. We also know that a slow type of processing for which attention is necessary and which is concentrated in the RH probably occurs for emotional words (Abbassi et al., 2011, for review). So, the question here is: what is the factor that is comparable to simulation, i.e., involves image reproduction, and for which the RH plays a critical role? According to literature, imagery has all these features (Holmes and Mathews, 2005, 2010; Holmes et al., 2008). Imagery is, in fact, a process that creates a mental image for the individual using different senses. Thus, it allows one to see, hear, smell, and feel different components of a situation (i.e., people, settings, actions, …) (Kosslyn et al., 2006). A distinguishing feature of emotional word processing is that image-based processing, in addition to including perceptual information, likely involves reactivated emotional properties based on the involvement of autobiographical memory. (see Cabeza and Nyberg, 2000, for a review). The suggestion is that autobiographical memory plays a pivotal role in the representation of emotional information (Conway, 1990).

In fact, the relationship between imagery and emotion is mediated by autobiographical memory. That is to say, a link between imagery and autobiographical memory is responsible for the emotional outcomes of image use. Holmes et al. (2008) attribute the more powerful impact of imagery on emotion, as compared to words, to three possible reasons: (1) the emotion system existed long before the language system^12^; (2) images share perceptual properties and details with actual experiences (Kosslyn et al., 2001); and (3) autobiographical memories, including emotional states experienced during interactions with the real world, are first stored in the form of images, not language. Images are therefore likely to be effective cues for reactivating emotional experiences. To be precise, imagery has a stronger emotional impact than purely linguistic forms because it has privileged access to the emotional experiences stored in autobiographical memory.

Research even suggests a causal relationship between imagery and emotion (Holmes et al., 2008); this implies that a more direct link exists between imagery and emotions, than between words and emotions. In this research, participants are given a combination of pictures and words conveying an emotional meaning (e.g., a picture of a flight of stairs and the word *fall* written beneath) and asked to rate their contents, without receiving any instructions concerning which modality to use. Results show that participants base their responses primarily on pictures, not words. Moreover, the more participants use images to respond, the more likely they are to report experiencing an emotional state.

Taking lateralization into consideration, the distinguishing feature of emotional word processing, i.e., imagery and the contribution of autobiographical memory should be responsible for the role that the RH play in this processing. Since the center of autobiographical memory retrieval is likely located in the RH (Tulving et al., 1994; Nyberg et al., 1996; Perecman, 2012; see Cabeza and Nyberg, 2000, for a review) and based on the aforementioned points, we can propose that the salient role of the RH in emotional word processing may relate to the retrieval of the contents of autobiographical memory surrounding past events and personal histories, which feeds imagery of emotional words. Pinpointing the role of the corpus callosum might also highlight the RH’s role in this process. Indeed, research shows that individuals with congenital absence of the corpus callosum produce language that contains almost no words denoting emotions (Turk et al., 2010). This deficit presumably causes the LH, which also has the role of generating language units such as words, to have reduced access to autobiographical memory contents in the RH. This deficit, which is called as *alexithymia*, has also been reported in patients with surgical disconnection of the cerebral hemispheres (Tenhouten et al., 1985a,b,c). Thus, there seems to be robust evidence supporting the salient role of the RH in deep processing of emotional words in which perceptual and emotional properties are involved, which coincides with an important role of the RH in autobiographical memory.

### The Suggested Framework: How Does Superficial vs. Deep Processing Occur?

One outcome of the above-mentioned superficial vs. deep processing types is that when we encounter an emotional word, we might access its semantically associated words, but not its perceptual and emotional properties. Following Barsalou et al. (2008), we believe that emotional words first access only semantically associated words, and that this process is focused mainly in the LH. When this process involves mental imagery and access to autobiographical memory contents, deeper processing occurs. This type of processing presumably occurs mainly in the RH and may be followed by experiencing a subjective emotional state.

For example, a word like “flower” might activate words like “rose,” “beautiful,” “fragrance,” and “branch.” When processing becomes deep due to, for instance, employing an explicit task or longer exposure duration of stimuli, the activated words lead to the recall of contents in autobiographical memory in which FLOWER can be found: this involves imagery. Therefore, depending on the individual’s memory content, a situation containing concepts like GARDEN, SPRING, PARK, WALKING, and NICE WEATHER may become active and the individual may see himself walking in a garden full of flowers in the spring, with nice weather, and finally perhaps feeling a pleasant emotional state.

On the unpleasant side, a word like “cancer” may activate words like “fear,” “bad,” “pain,” “disease,” and “death.” When processing becomes deeper, depending on the individual’s memory contents, a situation containing concepts like CHEMOTHERAPY, HOSPITAL, SURGERY, and FIGHT may be activated and the individual may see himself in a hospital room with a patient battling cancer and be left feeling an unpleasant emotional state. Obviously, not such an elaborated scenario is necessary or occurs all the time. So what may occur can be reactivation of only perceptual properties without emotional properties, or reactivation of emotional properties along with part of perceptual properties, etc.

Creating an emotional state, then, requires that autobiographical memory contents be activated and, consequently, that the emotional properties of an emotional concept are experienced; this is not possible unless imagery is involved. That is, activating semantically associated words and a superficial mode of processing does not lead to the experience of an emotional state *per se*. As well, activating perceptual properties does not create emotional feelings.

In sum, when emotional words are presented, the two systems of linguistic and imagery can become active. However, the linguistic system for which the LH is dominant seemingly operates automatically and, thus, peaks before the imagery system. As a result of this process emotional words likely establish a link with semantically associated words. When this superficial mode of processing is sufficient for adequate task performance, processing is supported mostly by the linguistic system and does not involve emotional feelings.

In contrast, when the linguistic system cannot complete a task on its own, or when there is more time to process emotional words, the imagery system may become involved. In this type of processing, the retrieval of past memories and situations containing these concepts occurs. This processing is deep and may be followed with subjective experiencing of an emotional state. We do not claim that processing in the RH always triggers an emotional state, but the level of processing in the RH should be deeper than in the LH and involves imagery. This latter stage, for which the RH is likely more responsible than the LH, is slow because more components (i.e., reactivation of perceptual and emotional properties) are involved.

## Conclusion

This paper investigates emotional words and the reason for fast vs. slow processing of these words, which occurs mainly in the LH and RH, respectively. Although we can use emotional words to convey emotional feelings, experiencing emotions may not be the primary outcome of using emotional words. This review suggests two systems of the linguistic and image-based (imagery) are involved in the processing of emotional words. As long as the processing involves mainly the linguistic system, emotional word processing does not necessarily result in emotional states.

Further research should be carried out using emotional words and tasks pinpointing a superficial vs. deep level of processing. Taking into consideration the few studies that have examined these two levels, using tasks such as word verification and word generation tasks should be helpful in revealing further aspects of this process. In addition, by using short vs. long exposure durations or tasks targeting superficial vs. deep processing, emotional word processing can be compared in the two hemispheres.

Thus, the level of emotional word processing in the RH should be deeper than in the LH and, thus, it is conceivable that the slow mode of processing in the RH adds certain qualities (reactivating perceptual and emotional properties) to the output.

## Conflict of Interest Statement

The authors declare that the research was conducted in the absence of any commercial or financial relationships that could be construed as a potential conflict of interest.

## Footnotes

1 This statement about the role of the LH and RH in early and later stage of emotional word processing, respectively, does not mean that processing occurs only in the LH or the RH. Instead, it implies that at these stages, the center of processing is in the LH or RH: there is relatively stronger activation in these respective hemispheres.

2 In this paper, we use *automatic* and *rapid* and also *attentional* and *slow* interchangeably.

3 A category of things distinguished by some common characteristic or quality (http://www.oxforddictionaries.com). Indeed, language is divided into content (meanings), form (i.e., rules, categories, structures), and use (pragmatics or the social use of language).

4 Throughout this paper, when we talk about words vs. concepts, quotation marks will be used to indicate words (e.g., “dog”) and uppercase will represent concepts (e.g., DOG). Italics are used to introduce new key technical terms or labels.

5 Although the terms *emotion* and *affect* are mostly used interchangeably, it is important not to confuse them. Affects are composed of responses involving respiratory system, blood flow changes, facial expressions, vocalizations, and viscera over which one has little control (Tomkins, 1995, p. 54). Affects, indeed, represent the way the body prepares itself for action in a given circumstances. Feelings and emotions, in contrast, are personal, biographical, and social, in the sense that every individual has his own set of sensations which are compared with previous experiences. While affects endow intensity to what we feel, feelings and emotions help us interpret and recognize the quality (i.e., pleasant, unpleasant) of our experiences and make our decision making activities more rational. Namely, the experience of emotions is highly subjective (Citron, 2012; Citron et al., 2014). For example, while we can speak broadly of certain emotions like anger, our own experience of this emotion is unique and probably multi-dimensional ranging from mild annoyance to blinding rage. Therefore, the term *emotion* seems to better explain subjective experiences that we refer to in this paper.

6 Properties or attributes perceived by emotional system. In general, emotional properties are considered as part of perceptual properties (e.g., Barsalou, 1999). Here, because the topic is directly related to a type of stimuli with emotional component (emotional words), we bring emotional properties into our attention, in order to be able to beter discuss their outcomes.

7 Since simulation is mostly considered to be an automatic process and, as we will see later on, deep processing of emotional words probably requires more attentional components due to the role that imagery plays in this processing (Pecher et al., 2009), we use the term *reactivation* as a substitute for *simulation* for deep processing of emotional words. We wish to be sufficiently cautious about the automatic vs. attentional nature of simulation and imagery, respectively.

8 This negative potential occurs in posterior scalp regions, 200–300 ms after word onset, for both negative and positive high-arousal words. It has been attributed to the arousal feature of emotional words and appears predominantly in the left occipitotemporal region (Kissler et al., 2007).

9 The emotional Stroop task is a version of the standard Stroop task (Stroop, 1935) in which participants are required to respond to the ink color of a color word while ignoring its meaning (e.g., the word *green* written in red ink). Since reading is an automatic process, naming the color in which a word is written requires the allocation of attention and, hence, causes longer naming times (the Stroop effect). Similarly, in the emotional Stroop task, naming the color of an emotional word takes longer than naming the color of a neutral word (the emotional Stroop effect). This effect reflects the fact that attention is captured by the emotional content of words (Williams et al., 1996).

10 Only the first two levels seem to be relevant to the topic of this paper.

11 Concept experts always compare simulation with imagery. While simulation is essentially an automatic process, attention is involved in imagery (Pecher et al., 2009). This conclusion is based on the results of research comparing these two processes (e.g., Wu and Barsalou, 2009). Participants in the imagery group were explicitly asked to form an image of a concept when responding to task items, while participants in the control group were required only to think about that concept and were not instructed to form an image. The claim that simulation occurs automatically arises from the results shown by the control group in this research, which presented similar results to the imagery group, implying that the control group also used imagery to respond.

12 Recall that Barsalou et al. (2008) also claimed that the simulation system evolved before the language system in human beings.
